# Supplementary material for: MetaRibo-Seq measures translation in microbiomes
Source: Nat Commun. 2020 Jun 29;11:3268. doi: 10.1038/s41467-020-17081-z (PMC7324362; doi:10.1038/s41467-020-17081-z)
Supplement: Supplementary file 10 — Supplementary Data 7 [file 41467_2020_17081_MOESM10_ESM.zip › File2/Confidence_High_Taxonomy/68497_out.krona.html]

Javascript must be enabled to view this page.

members
magnitude
magnitudeUnassigned
count
unassigned
taxon
rank

68497\_out

2

2
superkingdom
2

phylum
976
2

200643
class
2

171549
order
2

2
family
171550


SRS024388\_contig\_number\_contig-100\_11314.72029SRS047014\_contig\_number\_contig-100\_5819.157988
genus
239759
2
